# To explore the potential of LOXL2 as a biomarker in glioma and construct a genomic integrated clinical prognostic model

**DOI:** 10.3389/fonc.2025.1602475

**Published:** 2025-07-18

**Authors:** Hongjuan Wang, Qunli Li, Xiangtao Zheng, Weibiao Chen

**Affiliations:** ^1^ Cranial Ultrasound Chamber, Jinhua Traditional Chinese Medicine Hospital, Jinhua, Zhejiang, China; ^2^ Surgical Center, The Second Affiliated Hospital of Wenzhou Medical University, Yuying Children's Hospital, Wenzhou, Zhejiang, China

**Keywords:** LOXL2, glioma, clinical prognosis, immune infiltration, prognostic models

## Abstract

**Background:**

Glioma is a common invasive tumor of the central nervous system, and its pathological features significantly impair the quality of life of patients, with high mortality risk and easy recurrence. For glioma Lysine Oxidase 2(LOXL2), there are few reports in the scholarly literature. Based on the current situation of the insufficiency of current therapies, this study focuses on analyzing the biological function of LOXL2 in the occurrence and development of glioma by bioinformatics technology, and systematically evaluates the potential association between this molecular marker and the prognosis of patients. Through the integration of clinical data and molecular mechanism research, this study aims to provide a new theoretical basis for improving the diagnosis and treatment strategy of glioma.

**Methods:**

Integrated CCGA (exploratory) and TCGA (validation) cohorts. Analyzed LOXL2 expression patterns, GO/KEGG pathways, immune infiltration, single-cell distribution (scRNA-seq), and survival associations. Prognostic models were established via KM survival, COX regression, nomogram, and DCA.

**Results:**

LOXL2 overexpression correlated with higher glioma malignancy (P<0.001), particularly in IDH wild-type and 1p/19q non-codeleted subtypes (P<0.001). GO/KEGG revealed LOXL2 involvement in ECM remodeling. Immune analysis showed LOXL2 mediates macrophage-neutrophil immunosuppressive networks. scRNA-seq localized LOXL2 in tumor cells, stroma, and macrophages. High LOXL2 predicted worse overall survival (P<0.001). ROC-AUC for 1/3/5-year survival: CCGA: 0.817/0.897/0.925; TCGA: 0.793/0.776/0.730. Respectively, which proved that LOXL2 could be used as an independent prognostic indicator for glioma. Through the construction of nomogram and DCA model evaluation, the results indicate that LOXL2 has important translational value in the prognosis prediction of glioma.

**Conclusions:**

This study revealed that LOXL2 can be used as a potential biomarker in glioma and is correlated with clinical prognosis. LOXL2 may affect the dynamic balance of the tumor microenvironment by regulating the immune function of macrophages and neutrophils in the extracellular matrix (ECM). The prediction model was established based on CCGA clinical data and COX regression analysis of LOXL2 gene expression data, which provides a theoretical foundation for the development of LOXL2-targeted therapy and the construction of genomic integrated prognostic model.

## 1 Introduction

Gliomas are malignant lesions originating in the central nervous system, mainly involving the brain and spinal cord regions (1, 2). The disease has a high mortality and recurrence rate in clinical practice, which not only significantly impairs the quality of life of patients, but also becomes a prominent public health problem. Epidemiological surveys show that the annual prevalence of this disease is maintained at the level of 5–7 cases per 100–000 population. In addition, the treatment process involves high medical expenditure, posing a serious economic challenge to families and the medical insurance system (3). At present, clinical intervention strategies focus on surgical resection, radiotherapy and chemotherapy. However, due to the biological heterogeneity and easy recurrence of tumors, the clinical benefits of existing therapies are significantly limited (4–7). However, there are a variety of immunotherapy approaches for glioma, which can be combined with the influence of clinical factors on immunotherapy to help optimize individualized treatment regimens (8–11). Recently, bioinformatics studies have found that many molecules have the potential to be used as molecular markers of glioma, providing therapeutic targets for gene therapy and predicting tumor prognosis based on clinical survival data by machine learning algorithms (12–15). This study focused on the key molecule LOXL2, which plays an important regulatory role in the pathological process of glioma. Studies have confirmed that LOXL2 is significantly associated with tumor invasion and metastasis in a variety of malignant tumors (16). Especially in glioma, the expression intensity of LOXL2 is significantly correlated with the clinical outcome of patients, and its mechanism of action may involve biological processes such as tumor microenvironment remodeling and immune cell infiltration regulation (17, 18). These findings highlight the research value of LOXL2 as a novel biomarker. In this study, we integrated multi-dimensional bioinformatics techniques, including transcriptome data analysis, clinical prognostic parameter modeling and immune microenvironment analysis, to systematically elucidate the molecular mechanism of LOXL2 in glioma development and its clinical translational potential. The focus of this study is to verify the feasibility of LOXL2 as a diagnostic marker and evaluate its clinical application value in prognosis prediction. Through the integration and analysis of multi-omics data, this study establishes a systematic analysis framework, which provides a theoretical basis for the implementation of precision medicine strategies (19). With the in-depth study of the pathway of LOXL2, this molecule is expected to develop into a new therapeutic target and open up a new therapeutic approach for improving the clinical prognosis of glioma patients.

## 2 Methods

### 2.1 Acquisition of RNA sequencing data and clinical survival data

**Figure 1:**
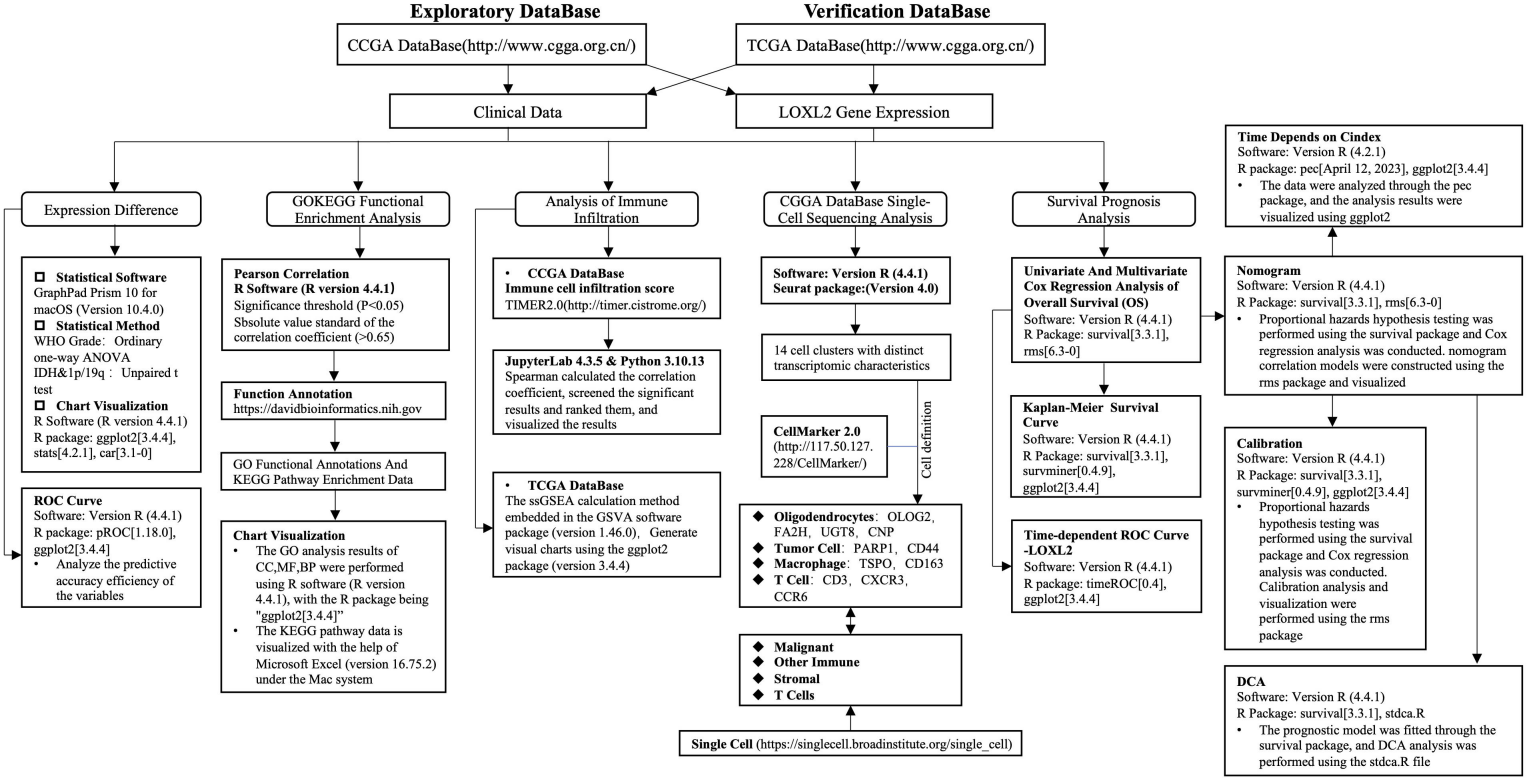
Analysis flow chart of this study. Included are the statistical methods, statistical software and chart visualization software used. It mainly includes gene expression difference, GO/KEGG enrichment analysis, immune infiltration analysis, single cell sequencing analysis, survival prognosis analysis and prognostic model construction and evaluation.

The clinical, RNA sequencing data and single cell sequencing data of all GBMLGG patients in this study were obtained from the CGGA website (http://www.cgga.org.cn/) (20). Validation set data downloaded from TCGA database (https://portal.gdc.cancer.gov) (21) and collates TCGA GBM and TCGA LGG project STAR process of RNAseq data and extract the TPM format of the data. This study adhered to the Declaration of Helsinki (2013 revision) and conformed to the publication guidelines provided by CCGA and TCGA. No studies on humans or animals by the authors were included in this study. The analysis flow chart of this study is shown in **Figure 1**.

### 2.2 Analysis of gene sequencing data and clinical data

The clinical data and survival data of CGGA dataset and TCGA dataset were visualized by R software (R version 4.4.1) with R package “pheatmap[1.0.12]” and “ggplot2[3.4.4]”. GraphPad Prism 10 for macOS (version 10.4.0) software was used for statistical analysis of sequencing data differences between CGGA dataset and TCGA dataset. Statistical methods: Ordinary one-way ANOVA was used for WHO Grade, Unpaired t test was used for IDH and 1p/19q. The expression differences between groups of sequencing results were completed by R software (R version 4.4.1), and the R package was “ggplot2[3.4.4]” for graphical visualization. The ROC curve of sequencing data was analyzed and visualized by R software (R version 4.4.1), R package: pROC[1.18.0], ggplot2[3.4.4].

### 2.3 GO/KEGG functional enrichment analysis of LOXL2 in glioma

In the analysis of CGGA and TCGA databases, the researchers used R language (version 4.4.1) to perform Pearson correlation calculation, and set the significance threshold (P<0.05) and the absolute value of correlation coefficient criterion (>0.65) for the selection of relevant genes. The gene sets that met the criteria were imported into the DAVID bioinformatics platform (version 6.8) for functional annotation, which selected standard gene nomenclature and limited the research object to the human genome. Through this process, gene ontology (GO) functional annotation and KEGG pathway enrichment data were successfully obtained, and the top six items with significance level (P<0.05) were selected for display. In terms of data presentation, the GO analysis results of cell component (CC), molecular function (MF) and biological process (BP) were visualized by R software (R version 4.4.1) with R package “ggplot2[3.4.4]”. KEGG pathway data were visualized using Microsoft Excel (version 16.75.2) under Mac system.

### 2.4 Immune infiltration analysis of LOXL2 in glioma

CCGA data set by TIMER2.0 (http://timer.cistrome.org/) will be graded to immune cells infiltrating CGGA expression data, and then use the Python (Python version number 3.10.13) extraction data LOXL2 expression and merged with immune score, Spearman correlation coefficient was used to calculate the correlation coefficient, and significant results were screened and ranked for visualization of the results. The immune infiltration analysis of TCGA database was performed in R language environment (version 4.4.1) using the ssGSEA calculation method embedded in GSVA software package (version 1.46.0) (Hanzelmann et al., 2013). The 24 immune cell marker gene sets published by Bindea in the journal Immunity were integrated to systematically evaluate the immune microenvironment characteristics of samples (22, 23). Then the ggplot2 toolkit (version 3.4.4) was used to complete the chart visualization.

### 2.5 Single-cell sequencing data analysis of LOXL2 in glioma

CCGA single-cell sequencing data sets were analyzed with R software (R version 4.4.1), cell clustering was performed with the Seurat package (version 4.0), and the results were visualized with the R package “ggplot2[3.4.4]”. Single-celled sequencing results of pretreatment process in accordance with the standards of previously reported methods, cell cluster similarity is through the same trait genes in clusters to define the number (24), first we define by CellMarker 2.0 (http://117.50.127.228/CellMarker/) cells, Oligodendrocyte:” *OLIG2, FA2H, UGT8, CNP* “; Tumor Cell: “*PARP1, CD44*”; Macrophage: “*TSPO, CD63*”; T Cell: “*CD3, CXCR3, CCR6*”. By UMAP dimensionality reduction, CGGA glioma single cells were divided into 14 cell clusters with clear transcriptomic characteristics. The 14 cell clusters were divided into four functional subgroups by unsupervised clustering analysis: Tumor Cell Area: cluster (2,3,5,6,7,10,17); Oligodendrocyte Area (0); Macrophage Area (1,4,8); T Cell Area (9,12), and the enrichment degree of LOXL2 in each cell cluster was observed by color depth on the UMAP map. Validation set us through Single Cell (https://singlecell.broadinstitute.org/single_cell). Enter the main page, The search strategy of single cell sequencing data set was “Metadata contains (species: Homo sapiens) AND (disease: glioma) AND (organ: Brain OR brain)”, and the target gene was selected as LOXL2. Cell cluster was defined as “Malignant, Stroma, T Cellsl, Other Immune”, which also had four functional cell subsets.

### 2.6 Prognostic analysis of clinical survival data and establishment of a prediction model

First, proportional hazards hypothesis test and Cox regression analysis were performed on CCGA and TCGA clinical survival data using R (4.2.1) version survival package [3.3.1], rms[6.3-0]. Variable screening strategy: if the sample in univariate satisfies the set P-value threshold, it will enter the multivariate Cox to construct the model. The P-value threshold is 0.1, and the univariate and multivariate COX analysis will be performed. Then, CGGA survival data were analyzed by GraphPad Prism 10 for macOS (version 10.4.0) software, logrank was used for statistical analysis, and the results were visualized by Python (Python version 3.10.13). TCGA survival data were completed using R software (R version 4.4.1), we used the R package “survival[3.3.1], survminer[0.4.9], ggplot2[3.4.4]”, and used the survival package to test the proportional hazards hypothesis and perform the fitting survival regression. The survminer package and ggplot2 package were used to visualize the results, and Cox regression was used for statistical analysis. In terms of prognostic model construction, time-dependent ROC analysis was performed by integrating CCGA and TCGA survival data and LOXL2 expression data by timeROC package pair, and the analysis results were visualized by ggplot2 to construct 1-year, 3-year and 5-year time-dependent ROC plots. The survival package was used to test the proportional hazards hypothesis and perform Cox regression analysis. The rms package was used to construct a nomogram related model and visualize it to construct a nomogram. The survival package was used for proportional hazard hypothesis testing and Cox regression analysis, and the rms package [6.3-0] was used for Calibration analysis and visualization. The survival package was used to fit the prognostic model, and the stdca R file was used for DCA analysis to construct the 1-year, 3-year, and 5-year prognosis DCA maps. The time-dependent Cindex was constructed by analyzing the data through the pec package. The C-index value of the survival model at different times was shown in the form of broken lines.

### 2.7 Statistical description

P < 0.05 was considered statistically significant. The following P values were considered: *P < 0.05, **P < 0.01, and ***P < 0.001, with statistical methods as described above.

## 3 Results

### 3.1 The potential value of LOXL2 as a biomarker for malignant progression of glioma

The baseline data of glioma patients divided into groups based on the expression level of LOXL2 in CCGA (**Table 1**) showed significant differences in key clinicopathological parameters: age distribution: patients in the LOXL2 high expression group were significantly older (45.7 ± 13.3 years *vs* 40.8 ± 10.0 years, p<0.001); Tumor grade: there was a significant difference in the distribution between groups (p<0.001); The patients with high LOXL2 expression were mainly WHO grade IV (35.5% *vs* 8.6%). The low LOXL2 expression group contained 29.6% WHO grade II tumors, which was significantly higher than 2.3% of the high LOXL2 expression group. The 1p/19q co-deletion was rare in the high expression group (0.7% *vs* 19.7%, p<0.001). IDH wild type was predominant in the high expression group (35.5% *vs* 10.9%, p<0.001). There was no significant difference in gender distribution between the two groups (p=0.193).

**Table 1:** Baseline data of the CCGA glioma database.

Characteristics	High expression of LOXL2	Low expression of LOXL2	P value
Age, mean ± sd	45.704 ± 13.288	40.842 ± 10.019	< 0.001
Gender, n (%)			0.193
Male	100 (32.9%)	89 (29.3%)	
female	52 (17.1%)	63 (20.7%)	
WHO grade, n (%)			< 0.001
WHO II	7 (2.3%)	90 (29.6%)	
WHO III	37 (12.2%)	36 (11.8%)	
WHO IV	108 (35.5%)	26 (8.6%)	
1p/19q codeletion, n (%)			< 0.001
Codel	2 (0.7%)	60 (19.7%)	
Non-codel	150 (49.3%)	92 (30.3%)	
IDH status, n (%)			< 0.001
Mutant	44 (14.5%)	119 (39.1%)	
Wildtype	108 (35.5%)	33 (10.9%)	

The analysis of baseline data of glioma patients based on TCGA database (**Table 2**) showed that the expression level of LOXL2 was significantly correlated with clinicopathological characteristics: patients in the high LOXL2 expression group were significantly older (51.7 ± 15.8 years *vs* 42.5 ± 13.3 years, p<0.001); There was no significant difference in gender distribution between groups (p=0.449). The difference in WHO grade distribution was extremely significant (p<0.001). The high expression group was mainly WHO grade IV; The 1p/19q co-deletion rate in the low expression group was 20.6%, which was 4.9 times higher than that in the high expression group (4.2%) (p<0.001). The proportion of IDH mutant in the low expression group was 41.7%, which was significantly higher than that in the high expression group (20.6%, p<0.001). The proportion of IDH wild type in the high expression group was 30.6%, which was 4.3 times that in the low expression group (7.1%).

**Table 2:** Baseline data of the TCGA glioma database.

Characteristics	High expression of LOXL2	Low expression of LOXL2	P value
Age, mean ± sd	51.698 ± 15.847	42.508 ± 13.334	< 0.001
Gender, n (%)			0.449
Male	189 (30.4%)	171 (27.5%)	
female	129 (20.8%)	132 (21.3%)	
WHO grade, n (%)			< 0.001
WHO II	50 (8.1%)	172 (27.7%)	
WHO III	122 (19.6%)	123 (19.8%)	
WHO IV	146 (23.5%)	8 (1.3%)	
1p/19q codeletion, n (%)			< 0.001
Codel	26 (4.2%)	128 (20.6%)	
Non-codel	292 (47%)	175 (28.2%)	
IDH status, n (%)			< 0.001
Mutant	128 (20.6%)	259 (41.7%)	
Wildtype	190 (30.6%)	44 (7.1%)	

**Figure 2:**
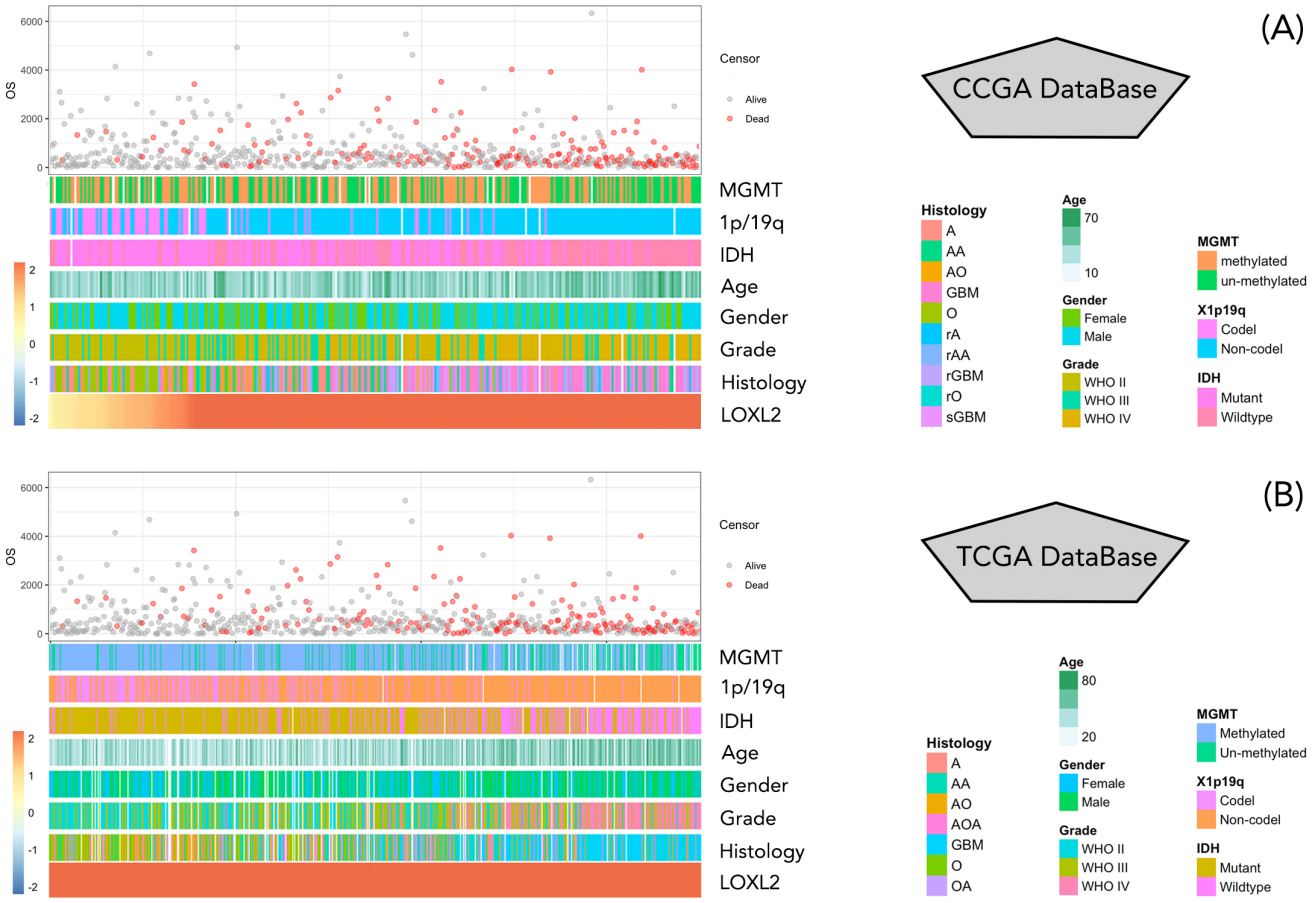
**(A, B)** represent the display of clinical data and survival data of patients in CCGA database and TCGA database, respectively. The clinical data information included MGMT, 1p/19q, IDH, Age, Gender, Grade, Histology, and were arranged from low to high according to the value of LOXL2 expression data. Patient survival information is in days, red represents survival, gray represents death.

**Figure 3:**
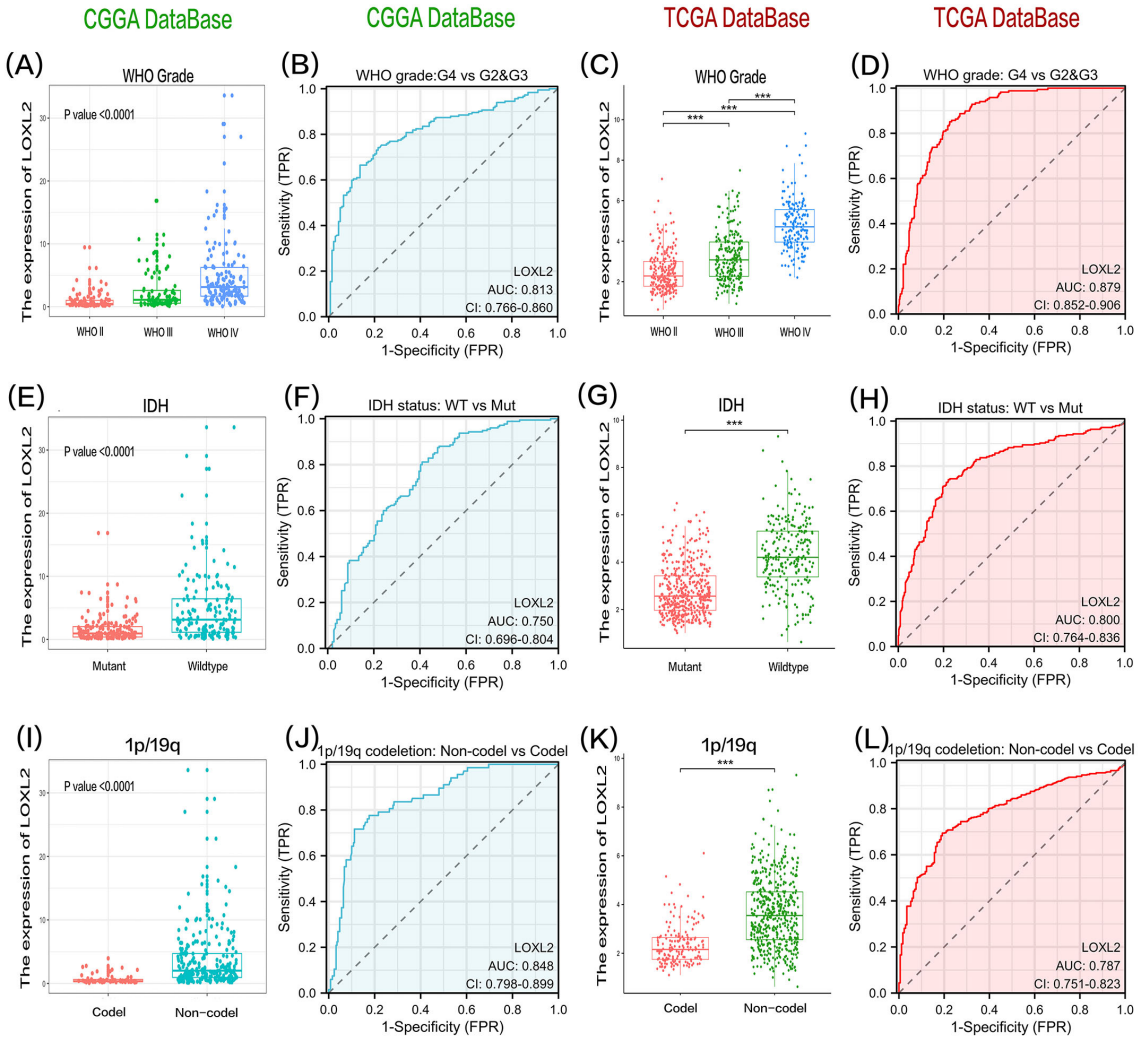
The data of group **(A)** and group **(C)** showed that the expression level of LOXL2 in high-grade glioma tissues showed statistically significant up-regulation in CGGA and TCGA two independent databases. The significance of the difference was evaluated by one-way analysis of variance. The results of group **(E, G)** showed that the expression of LOXL2 in glioma samples with IDH wild-type was significantly higher than that in the cases with IDH gene mutation, and the difference between the two groups was verified by unpaired t test. Analysis of groups **(I, K)** revealed that the expression intensity of LOXL2 in gliomas lacking 1p/19q co-deletion was significantly higher than that in the control group, and the statistical difference was also verified by unpaired t test. **(B, D, F, H, J, L)** are the ROC curves of grouping variables, G2G3 *vs* G4 in WHO Grade group, Mutant *vs* Wildtype in IDH group, and Codel vs Non-Codel in 1q/19q group. The “***” in **(C,G,K)** indicates a p-value < 0.001.

The clinical data and survival data of patients in CCGA and TCGA datasets are shown in **Figure 2**. The gene expression data are shown by grouping differential expression (**Figures 3A, C, E, G, I, K**). There were significant differences in the expression of LOXL2 in WHO Grade, isocitrate dehydrogenase (IDH) mutation and 1p/19q deletion groups between CCGA and TCGA datasets (P<0.0001). ROC curve showed that WHO Grade G2G3&G4 (CGGA: 0.813; TCGA: 0.879), IDH Mutant& Wildtype (CCGA: 0.750; TCGA: 0.800), 1p/19q Codel& non-codel (CGGA: 0.848; TCGA: 0.787) (**Figures 3B, D, F, H, J, L**). This analysis revealed that high expression of LOXL2 was strongly correlated with advanced pathological characteristics of glioma, IDH wild type and 1p/19q non-codeletion, suggesting its potential value as a biomarker for malignant progression of glioma.

### 3.2 LOXL2 exerts its biological function through extracellular matrix in glioma

**Figure 4:**
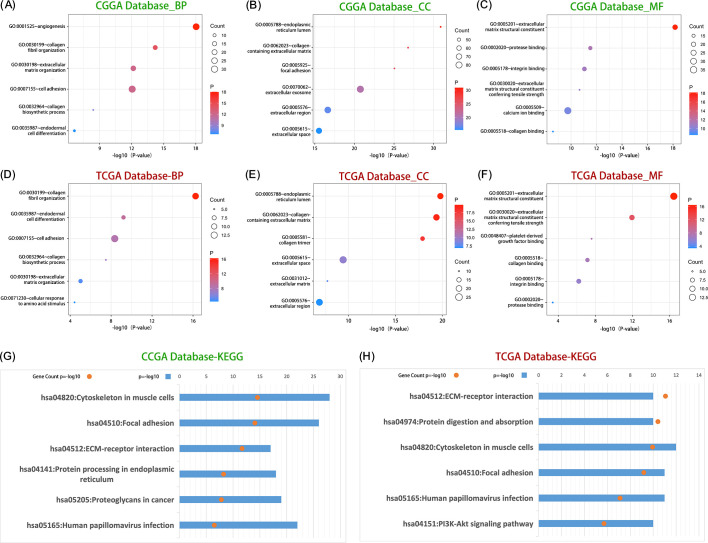
LOXL2 was significantly associated with the regulation of ECM processes in glioma. Group **(A–C)** shows the distribution of core biological processes (BP), cell composition (CC) and molecular function (MF) in which LOXL2 was significantly involved in the CGGA database. The **(D–F)** plots revealed the dominant role of LOXL in regulating biological process (BP), cellular component (CC) and molecular function (MF) in TCGA database. Group **(G)** data showed the enrichment of KEGG signaling pathways related to LOXL2 in CGGA database, and group **(H)** systematically showed the regulatory mechanism of KEGG pathway network involved in LOXL2 in TCGA database.

In order to explore the biological functions related to LOXL2, Pearson correlation analysis (|R| > 0.65, P < 0.05) was used to screen the genes with the highest correlation with LOXL2 in TCGA and CGGA databases. GO and KEGG analyses were performed based on the above gene sets. Through functional enrichment analysis of the cross-dataset (CCGA and TCGA) of LOXL2 in glioma, we found that its core functions were significantly focused on extracellular matrix (ECM) dynamic remodeling and tumor invasion microenvironment regulation. Among the biological processes (BP) (**Figures 4A, D**), LOXL2 strongly drives angiogenesis, collagen fiber assembly and ECM remodeling. Cell localization (CC) showed that LOXL2 was mainly enriched in collagenetic ECM structures, focal adhesion and ER lumen (**Figures 4B, E**). At the molecular function (MF) level (**Figures 4C, F**), LOXL2 specifically binds to collagen and integrin, and endows the ECM with tensile strength, providing a mechanical shelter microenvironment for glioma stem cells. KEGG pathway analysis further revealed that LOXL2 may activate the FAK/PI3K-Akt signaling axis through ECM-receptor interaction (**Figures 4G, H**), suggesting its potential role in glioma immune escape.

### 3.3 LOXL2 may regulate the immune function of glioma through the cooperation of macrophages and neutrophils

**Figure 5:**
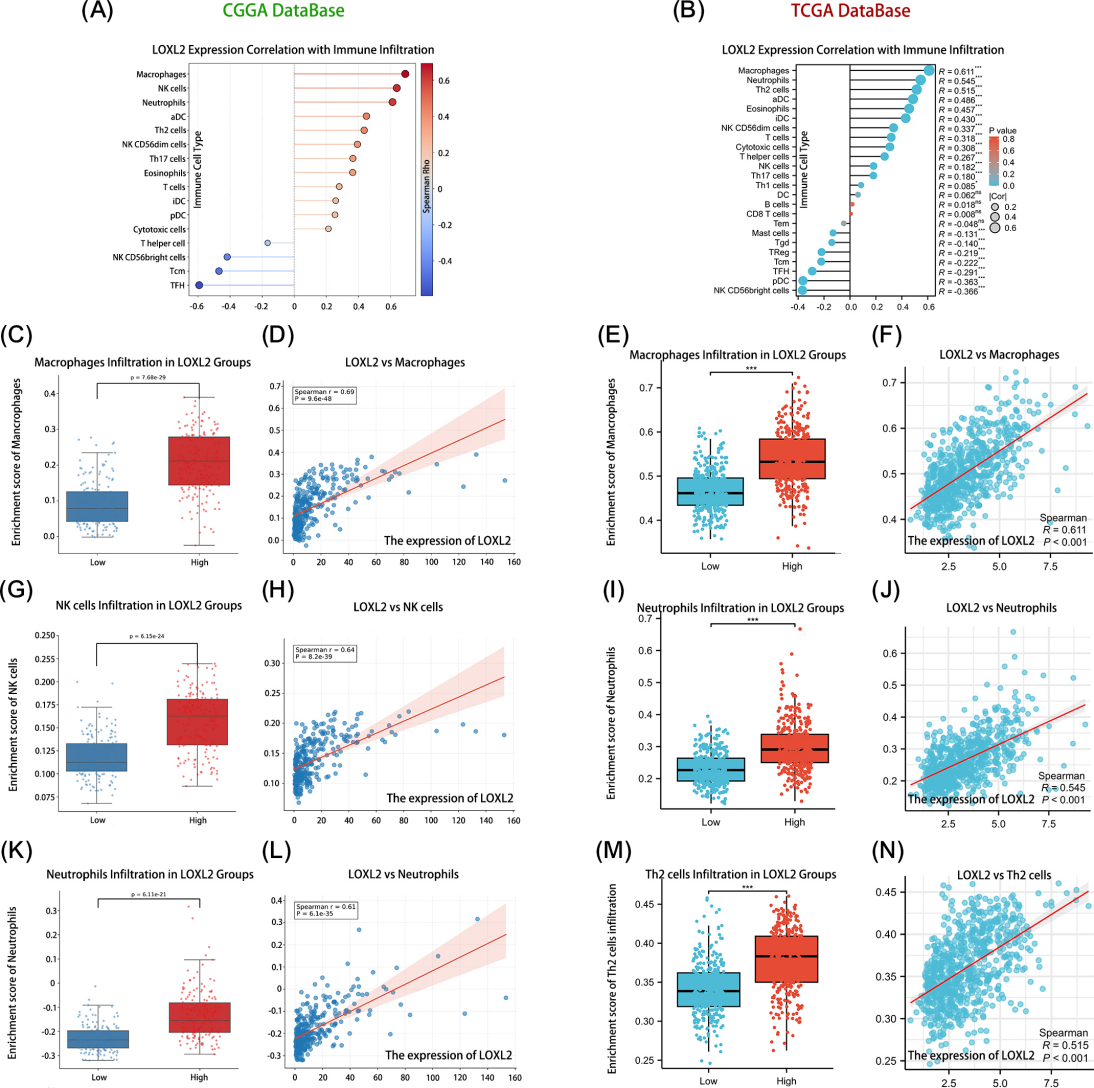
Shows that LOXL2 plays an important role in glioma immune regulation. The correlation between the core variables and the immune infiltration matrix data was evaluated. Subplots **(A, B)** show the expression correlation between LOXL2 and the relative abundance of immune cells in the CCGA and TCGA glioma databases, respectively. Subplots **(D, H, L, F, J, N)** revealed the quantitative relationship between the expression level of LOXL2 and the infiltration degree of immune cells such as macrophages, NK cells, neutrophils, and Th2 cells. Subplots **(C, G, K, E, I, M)** focused on the dynamic changes in the expression of LOXL2 and the infiltration of immune cell subsets in the high and low risk groups. The “***” in **(E, I, M)** indicates a p-value < 0.001.

In order to explore the immune infiltration function related to LOXL2, the data analysis results were visualized (**Figures 5A, B**). The size of each circle represents the correlation between the enrichment degree of immune cells and LOXL2, and the color of the circle represents the P value. The distance between the circle and the baseline also indicates the correlation between the enrichment of the immune cell and LOXL2, which is the same as the significance of the size of the circle. Subsequently, the top 3 immune cells with the strongest correlations and significant differences in p-values were selected (**Figures 5C–N**). The expression of LOXL2 was strongly positively correlated with the infiltration of macrophages (CGGA: R=0.69, TCGA: R=0.61) and neutrophils (CGGA: R=0.61, TCGA: R=0.55) (all P<0.001). There was a significant correlation between CCGA-NK cell infiltration (R=0.64, P<0.001), suggesting that LOXL2 may affect immune surveillance through NK cell activation pathway. Tcga-nk cell correlation (R=0.52, P<0.001), suggesting that LOXL2 is involved in the regulation of Th2-type immunosuppressive microenvironment. Through immune infiltration analysis, we found that LOXL2 may regulate the immune function of glioma through the cooperation of macrophages and neutrophils.

### 3.4 LOXL2 is involved in the regulation of malignant phenotype of tumor cells and the function of immune cells through stromal cell remodeling

**Figure 6:**
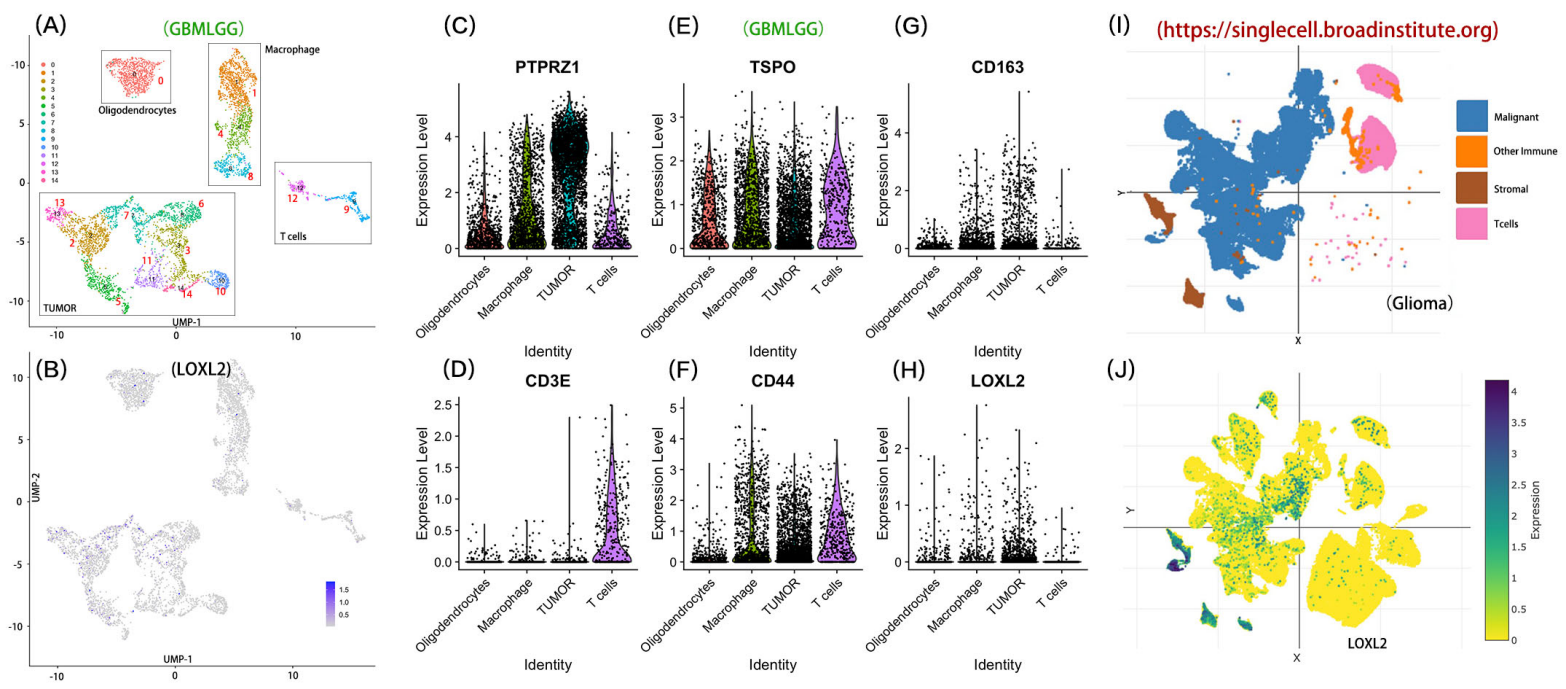
Distribution characteristics of LOXL2 in immune cells and tumor cells. Section **(A)** shows the four functional categories: the tumor cell region contains clusters (2,3,5,6,7,10,17); Oligodendrocyte region corresponds to cluster 0; The macrophage area covered clusters 1, 4, and 8. The T-lymphocyte region was composed of clusters 9 and 12. Section **(B)** shows the expression level of LOXL2 at the single-cell level in CCGA glioma samples. **(C-H)** part compares the tumor cells, oligodendrocytes, macrophages and T lymphocytes area LOXL2 and characteristic of molecular markers in (including PTPRZ1, TSPO, CD163, CD3E, CD44) differences in expression patterns. Sections **(I, J)** verified the enrichment of LOXL2 expression in glioma tissues by single-cell sequencing data, respectively.

We through the single-cell transcriptome analysis reveals LOXL2 based in glioma regulatory role in the immune microenvironment CCGA glioma single-celled sequencing data, through the UMAP dimension reduction could be divided into 14 CGGA glioma single transcriptome study characteristics of clear cell clusters. The 14 cell clusters were divided into four functional subgroups by unsupervised clustering analysis: Tumor Cell Area: cluster (2,3,5,6,7,10,17); Oligodendrocyte Area (0); Macrophage Area (1,4,8); T Cell Areas (9,12) (**Figure 6A**). The expression of LOXL2 in the target Cluster was 3.55 times higher than that in the control group. 28.7% of the cells in Cluster 7 expressed LOXL2, while only 13.6% of the cells in the control group expressed LOXL2, with a 2.1-fold difference. LOXL2 was specifically highly expressed in cluster 7, which was located in the Tumor Cell Area defined by us and may be a key marker, suggesting that LOXL2 is closely related to tumors (**Figure 6B**). According to the grouping difference (**Figures 6C–H**), most of LOXL2 was enriched in the Aacrophage Area and Tumor Area. By single database SingleCell (https://singlecell.broadinstitute.org) analysis of the independent validation set (**Figures 6I, J**), we observed a similar population distribution characteristics, four types of cells Malignant Cells, Stromal cells, Other Immune cells and T Cells. Across data set analysis shows that LOXL2 genes show significant cell type specific expression pattern: in the CCGA data mainly occurred in macrophages and tumor cells, while in validation specificity high expression in malignant cells and stromal cells. This cross-platform and repeatable expression pattern suggests that LOXL2 may affect the tumor microenvironment through dual mechanisms, participate in the regulation of tumor cell malignant phenotype and affect the function of immune cells through stromal cell remodeling.

### 3.5 LOXL2 as glioma independent prognostic markers, and the evaluation of the clinical prognosis prediction model is established and the model

Univariate and multivariate Cox regression analysis of overall survival (OS) showed that the univariate and multivariate Cox regression analysis of Age, Grade, IDH and 1p/19q in CCGA and TCGA datasets were p<0.001, suggesting that in Cox regression analysis, LOXL2 expression was an independent prognostic factor independent of known prognostic factors, including WHO grade, age at diagnosis, IDH mutation and 1p/19q deletion. These findings suggest that LOXL2 in CGGA and TCGA database are independent prognostic factors (**Tables 3**, **4**).

**Table 3:** Univariate and multivariate Cox regression analysis of overall survival (OS) in CCGA database.

Characteristics	Total (N)	Univariate analysis		Multivariate analysis	
		Hazard ratio (95% CI)	P value	Hazard ratio (95% CI)	P value
Age	313	1.033 (1.020-1.046)	**< 0.001**	1.011 (0.999-1.024)	0.073
Gender	313				
Male	197	Reference			
female	116	1.063 (0.809-1.397)	0.660		
Grade	309				
WHO II	98	Reference		Reference	
WHO III	74	3.498 (2.287-5.348)	**< 0.001**	3.404 (2.207-5.249)	**< 0.001**
WHO IV	137	8.902 (5.996-13.215)	**< 0.001**	6.153 (4.012-9.436)	**< 0.001**
IDH	312				
Mutant	167	Reference		Reference	
Wildtype	145	2.821 (2.137-3.724)	**< 0.001**	0.908 (0.653-1.264)	0.569
1p/19q	305				
Codel	62	Reference		Reference	
Non-codel	243	5.890 (3.610-9.609)	**< 0.001**	4.026 (2.387-6.788)	**< 0.001**

Univariate COX regression analysis showed that the P values of age, WHO grade, IDH, and 1p/19q were less than 0.05. Multivariate COX regression analysis showed that P values of WHO grade and 1p/19q were both < 0.05, suggesting that these variables were potential factors affecting survival prognosis.

Bold font indicates that the association between the variable and survival risk is statistically significant, meaning that the effect of the variable on survival time is significant (significance level α=0.05).

**Table 4:** Univariate and multivariate Cox regression analysis of overall survival (OS) in TCGA database.

Characteristics	Total(N)	Univariate analysis		Multivariate analysis	
		Hazard ratio (95% CI)	P value	Hazard ratio (95% CI)	P value
Age	603	1.075 (1.063-1.088)	**< 0.001**	1.046 (1.031-1.061)	**< 0.001**
Gender	603				
Male	349	Reference			
female	254	0.999 (0.742-1.345)	0.997		
Grade	603				
WHO II	213	Reference		Reference	
WHO III	238	3.257 (1.988-5.337)	**< 0.001**	2.173 (1.291-3.658)	**0.003**
WHO IV	152	20.125 (12.181-33.248)	**< 0.001**	3.764 (2.017-7.025)	**< 0.001**
IDH	597				
Mutant	373	Reference		Reference	
Wildtype	224	11.071 (7.772-15.771)	**< 0.001**	3.079 (1.836-5.161)	**< 0.001**
1p/19q	598				
Codel	149	Reference		Reference	
Non-codel	449	4.541 (2.671-7.719)	**< 0.001**	1.943 (1.049-3.599)	**0.035**

Univariate and multivariate COX regression analysis showed that the P values of age, WHO grade, IDH, and 1p/19q were all less than 0.05, suggesting that these variables were potential factors affecting survival prognosis.

Bold font indicates that the association between the variable and survival risk is statistically significant, meaning that the effect of the variable on survival time is significant (significance level α=0.05).

**Figure 7:**
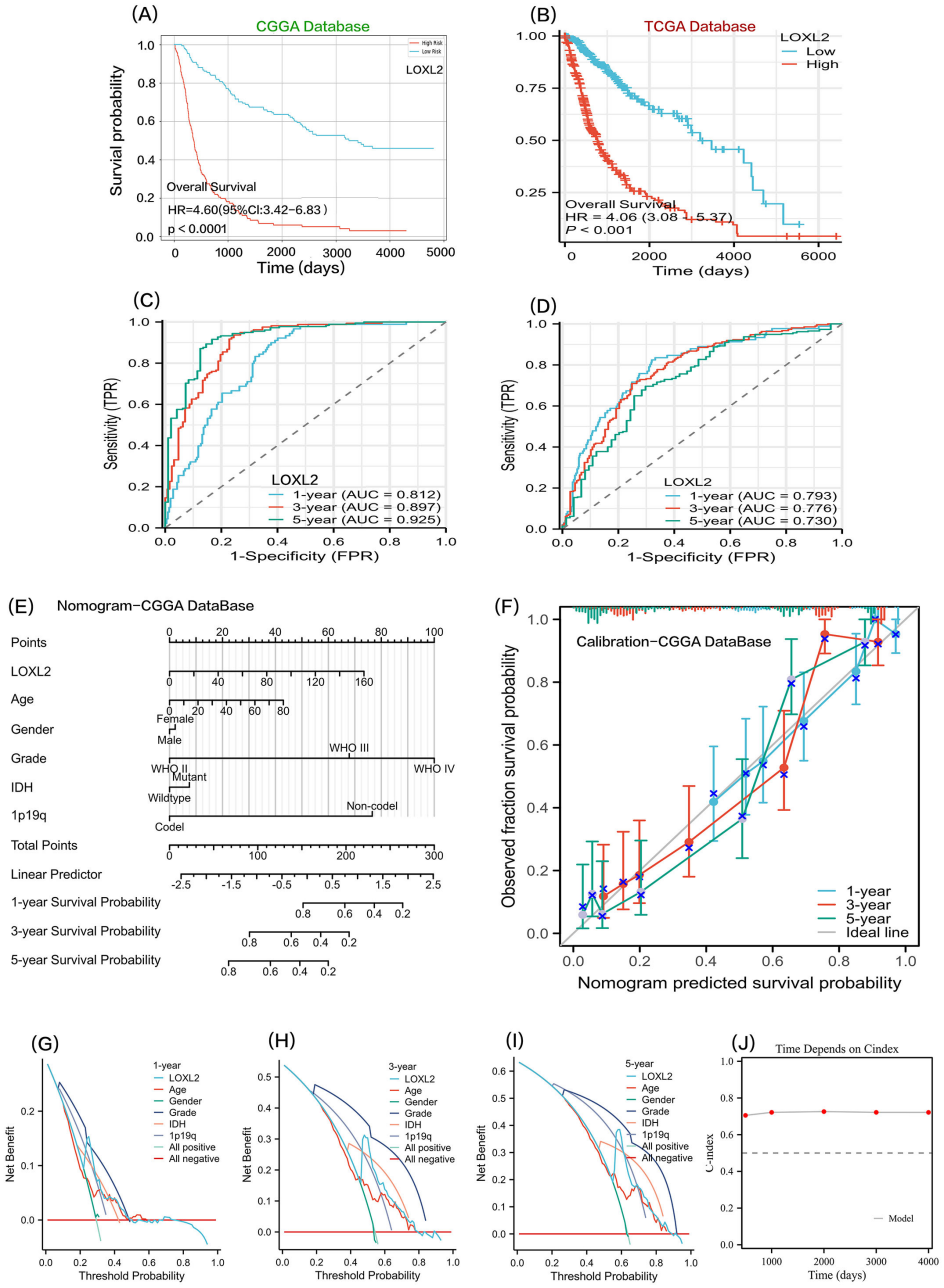
Sections **(A, B)** show the Kaplan-Meier survival curve analysis of LOXL2 gene expression levels in CGGA and TCGA glioma datasets. Sections **(C, D)** show time-dependent ROC curve evaluation of LOXL2 biomarkers for CGGA and TCGA glioma cohorts. **(E, F, G, H, I)** all analyses were based on the COX multivariate regression model of CCGA clinical data and LOXL2 expression data. **(E)** a prognostic nomogram model was constructed. **(F)** is the prognostic Calibration plot, depicting the difference between the predicted probability and the actual probability corresponding to the model at different time points. **(G–I)**, To evaluate the clinical utility of the model or patient benefit, and to construct decision curve analysis (DCA) at 1 year, 3 years, and 5 years. **(J)** time-dependent Cindex: The C-index values of the survival model at different times are shown in the form of broken lines.

To explore the prognostic predictive value of LOXL2 in glioma patients, Kaplan-Meier analysis and Cox proportional hazard model analysis were performed based on CGGA and TCGA database (**Figures 6A, B**). In CGGA database, the overall survival of patients with high expression of LOXL2 was significantly shorter than that of patients with low expression of LOXL2, HR=4.60 (Cl: 3.42-6.83), with statistical significance (P<0.001). In addition, the prognostic value of LOXL2 was also verified in TCGA database, with HR=4.06 (Cl: 3.08-5.87), which was statistically significant (P<0.001). The time-dependent ROC curve showed that CCGA 1-year AUC=0.817, TCGA 1-year AUC=0.793, CCGA 3-year AUC=0.897, TCGA-3 year AUC=0.776, TCGA 5-year AUC=0.730, CCGA 5-year AUC=0.925 (**Figures 7 C, D**).

We combined CCGA clinical survival data and LOXL2 expression data. On the basis of multivariate regression analysis, a prognostic Nomogram was established, and multiple predictors were integrated by using graduated line segments and plotted on the same plane according to a certain proportion to express the relationship between each predictor variable in the prediction model. A prognostic nomogram and a prognostic calibration curve were constructed (**Figures 7E, F**). In order to benefit patients with clinical utility or evaluation model, we also built a 1 year, 3 years, 5 years of decision-making curve analysis (DCA)(**Figures 7G–I**). the value of the note is LOXL2 DCA curves in the threshold probability (Pt) is about 0.25 (1 year), 0.50 (3 years), There was a significant peak of receiving treatment benefit at 0.62(at 5 years). The time-dependent C-index was balanced around 0.7 at 500, 1000, 2000, 3000, and 4000 days, indicating that LOXL2 has important translational value in predicting the prognosis of glioma.

## 4 Discussion

Glioma is the most common and deadly cancer of the central nervous system (9). Traditional therapies usually include surgical resection, radiotherapy, and temozolomide chemotherapy, but in general, the efficacy in patients with glioblastoma is not ideal. Although many patients with other cancers have benefited from immunotherapy, the clinical efficacy of immunotherapy in patients with glioblastoma remains disappointing. Exploring the molecular mechanisms of glioma-induced immunosuppression and developing novel therapies that exploit these mechanisms are critical for clinical management and prognosis.

Studies have shown that lysine oxidase 2 (LOXL2) plays an important role in a variety of cancers, and its expression level is closely related to tumor invasion, metastasis and clinical prognosis (25). However, there are few relevant literatures on LOXL2 in glioma. In this study, we performed a comprehensive analysis of LOXL2 in glioma by using bioinformatics technology, including clinical samples and gene expression data from different countries.

In this study, based on the mRNA-seq data of glioma patients from CGGA and TCGA databases, the expression levels of LOXL2 in different pathological types of gliomas were analyzed. The results showed that LOXL2 was enriched in more malignant glioma subtypes and showed specific molecular characteristics in IDH wild type and 1p/19q uncodeleted subtypes. Based on GO functional enrichment and KEGG analysis, we found that LOXL2 may exert its biological functions through the extracellular matrix (ECM). Immune infiltration analysis showed that LOXL2 was closely related to macrophages, neutrophils and NK cells in glioma. Single cell sequencing data analysis showed that LOXL2 was mainly enriched in tumor cells, macrophages and stromal cells. Finally, by analyzing the clinical survival data, we revealed that there was a difference in the survival time of patients between the high and low risk groups, and we established a machine learning model for LOXL2 to predict clinical prognosis.

In summary, this study comprehensively analyzed the potential of LOXL2 as a glioma biomarker. LOXL2 may play an immunomodulatory role in the extracellular matrix (ECM) through macrophages and neutrophils, and prognostic analysis of clinical survival time through the prediction model. A non-invasive prognostic prediction model was constructed. This study provides a theoretical basis for the immunophenotyping of gliomas and the development of combined treatment strategies, which has important translational medicine value. It is hoped that future research will focus on LOXL2 to achieve the goal of individualized treatment.

## Data Availability

Publicly available datasets were analyzed in this study. This data can be found here: https://singlecell.broadinstitute.org.
